# Evaluation and comparison of oral endotracheal tube depth prediction formulas in children with scoliosis: a retrospective study

**DOI:** 10.3389/fped.2026.1828762

**Published:** 2026-06-01

**Authors:** Yangyang Zhang, Peng Gao, Qingshui Zheng, Bo Zhu

**Affiliations:** Department of Anesthesiology, Peking Union Medical College Hospital, Chinese Academy of Medical Sciences and Peking Union Medical College, Beijing, China

**Keywords:** children, endotracheal intubation depth, oral endotracheal intubation, pediatric anesthesia, scoliosis

## Abstract

**Background:**

Children with scoliosis exhibit unique anatomical characteristics which challenge the accurate prediction of endotracheal tube (ETT) depth.

**Objective:**

To evaluate commonly used pediatric ETT depth prediction methods in children with scoliosis.

**Methods:**

This single-center retrospective study included children undergoing posterior thoracolumbar corrective surgery. Univariate and multivariable linear regressions identified independent predictors of intubation depth. Four conventional formulas (Age/2 + 12, ID × 3, Height/10 + 5, Weight/2 + 8) and x-ray-measured tracheal length were evaluated using Bland-Altman analysis and mean absolute error (MAE).

**Results:**

197 children aged 2–12 years were analyzed. Height correlated most strongly with documented ETT depth (Pearson's *r* = 0.832, *R*^2^ = 0.692). The Height/10 + 5 formula had the smallest MAE (0.996 cm) and highest safe proportion (72.08%; |predicted–actual|≤0.15×tracheal length). Other conventional formulas had larger MAEs (1.713∼5.418 cm). Regression-derived formulas improved performance but did not surpass Height/10 + 5. Tracheal length alone had limited predictive power (*R*^2^ = 0.499).

**Conclusion:**

In children with scoliosis, the Height/10 + 5 formula showed the closest agreement with documented ETT depth. Regression-derived formulas require external validation. x-ray-measured tracheal length should be used only adjunctively.

## 1 Introduction

Endotracheal intubation constitutes a core technology in both general anesthesia and critical care medicine. The accuracy of intubation depth is directly associated with patient safety and clinical outcomes. Children, particularly those with congenital or acquired anatomical abnormalities, exhibit distinct airway developmental and structural characteristics compared to adults (1). Consequently, the management of tracheal intubation depth in this population poses greater challenges and necessitates enhanced accuracy in depth prediction.

Scoliosis is defined as a complex three-dimensional spinal deformity characterized by lateral curvature, sagittal plane lordosis or kyphosis, and vertebral rotation. Children with scoliosis not only present with spinal curvature but also frequently exhibit thoracic deformities, reduced lung volume, and potential associated congenital syndromes. Collectively, these factors result in substantial alterations in airway anatomy (2, 3). However, most commonly used prediction formulas for pediatric endotracheal intubation depth are derived from studies involving healthy children with normal growth and development. Sufficient evidence regarding the applicability of these formulas to children with scoliosis and anatomical abnormalities remains lacking (4).

This study is the first to systematically evaluate and compare tracheal intubation depth formulas in children with scoliosis, a population with complex airway and thoracic anatomical abnormalities. We retrospectively analyzed clinical data from children undergoing scoliosis surgery at our institution. We aimed to evaluate the applicability of four commonly used pediatric endotracheal tube (ETT) depth prediction formulas—Age/2 + 12, ID × 3, Height/10 + 5, and Weight/2 + 8—as well as tracheal length measured on X-ray in this specific population (5–8). We further compared the predictive accuracy of these methods and explored the feasibility of establishing a more individualized intubation depth prediction model for this cohort. This study is expected to provide anesthesiologists with more evidence-based, tailored recommendations for determining intubation depth in children, while bridging the current evidence gap in pediatric anesthesia practice, thereby carrying important clinical and research implications.

## 2 Methods

### 2.1 Study design and population

This single-center, retrospective study was approved by the Ethics Committee of Peking Union Medical College Hospital (approval number: I-26PJ0380). Due to the retrospective nature of data collection, the requirement for informed consent was waived in accordance with ethical guidelines. Electronic medical records of children aged 2–12 years with a diagnosis of scoliosis who underwent posterior thoracolumbar corrective surgery under general anesthesia with orotracheal intubation between April 2022 and September 2025 were retrospectively analyzed.

### 2.2 Inclusion and exclusion criteria

Inclusion Criteria: 1. Orotracheal intubation performed during the surgical procedure; 2. Age ranging from 2 to 12 years; 3. American Society of Anesthesiologists (ASA) physical status classification I–III.

Exclusion Criteria: 1. Missing key clinical data or obvious errors in medical records; 2. History of prior tracheal surgery; 3. Known congenital airway malformations unrelated to scoliosis.

### 2.3 Data collection

The following data were extracted from anesthesia records and the hospital's Picture Archiving and Communication System (PACS):

Patient Demographics: age (years), sex, height (cm), weight (kg) and body mass index (BMI, kg/m^2^).

Intubation Details: Internal diameter (ID, mm) of the cuffed ETT utilized. The initial ETT depth was set at the second black circumferential line from the ETT at the glottal level, a routine institutional practice independent of any prediction formula. The final ETT depth (cm) at the upper incisor or gingiva margin was documented by the attending anesthesiologist after confirmation by capnography and auscultation.

Radiographic Measurement: Tracheal length was measured on posteroanterior (PA) upright chest radiographs by a single investigator blinded to clinical data, using the PACS ruler at 200% magnification. Tracheal length was defined as the linear distance from the vocal cord plane to the carina. A standardized two-step method was employed for landmark identification: (1) On lateral chest radiographs, the vertebral body corresponding to the vocal cord plane was identified; (2) This vertebral level was then referenced on the corresponding PA chest radiograph, with the horizontal line at that level designated as the vocal cord plane (9). To evaluate measurement reliability, 20% of randomly selected radiographs (*n* = 39) were re-measured by the same primary investigator (intra-observer) and independently measured by a second blinded investigator (inter-observer).

### 2.4 Statistical analysis

The documented ETT depth was used as the reference standard. Predicted depths were calculated using four commonly used pediatric ETT depth formulas (Age/2 + 12, ID × 3, Height/10 + 5, Weight/2 + 8). Statistical analysis was performed using SPSS Statistics (Version 26.0; IBM Corp., Armonk, NY, USA).

Intraclass correlation coefficient (ICC) for absolute agreement (two-way mixed model) was used to assess the radiographic measurement reliability.

Pearson correlation coefficient (*r*) was used to assess the linear association between predicted depths (and other continuous variables) and documented depth, with interpretation as follows: *r* < 0.5 (weak correlation), 0.5 ≤ *r* < 0.7 (moderate correlation), and *r* ≥ 0.7 (strong correlation). The coefficient of determination (*R*^2^) derived from simple linear regression was used to quantify the proportion of variance in documented ETT depth explained by predicted depth.

A multivariable linear regression analysis was performed using a stepwise selection method. Variables with *P* < 0.10 in univariate analysis were entered into the multivariable model. Multicollinearity was assessed using variance inflation factor (VIF), with VIF ＞ 10 indicating significant collinearity. The adjusted *R*^2^ was reported to reflect the proportion of variance explained by the final model.

Bland-Altman analysis was performed to evaluate the concordance between predicted and documented ETT depths, reporting the mean difference (bias) and 95% limits of agreement [95% LoA: mean difference ± 1.96 standard deviations (SD) of the differences]. Predictive accuracy was further quantified using mean absolute error (MAE) and root mean square error (RMSE). A two-sided *p*-value < 0.05 was considered statistically significant.

A prediction was considered clinically safe if |predicted depth—documented depth| ≤ 0.15 × tracheal length. The multiplier 0.15 was derived from the clinically recommended 1-2 cm safety margin (median 1.5 cm) divided by the mean tracheal length in our cohort (∼9.8 cm).

## 3 Results

### 3.1 Patient characteristics

A total of 197 children aged 2–12 years were enrolled in this study, including 104 females and 93 males. The mean age was 8.04 ± 3.26 years, and the mean body mass index (BMI) was 17.18 ± 3.72 kg/m^2^ (Table 1). Reliability analysis of tracheal length measurements showed excellent intra-observer (ICC = 0.94, 95% CI: 0.90–0.97) and inter-observer (ICC = 0.89, 95% CI: 0.83–0.93) agreement.

**Table 1:** General information of the study population.

Variable	Total (*N* = 197)
Female patients, *n* (%)	104 (52.8%)
Age (years), mean ± SD	8.04 ± 3.26
Height (cm), mean ± SD	127.41 ± 20.94
Weight (kg), mean ± SD	29.64 ± 14.51
BMI (kg/m^2^), mean ± SD	17.18 ± 3.72
ID (mm), median (range)	5.5 (4–7)
Tracheal length (cm), mean ± SD	9.77 ± 2.10
Documented ETT depth (cm), mean ± SD	17.63 ± 2.30

BMI, body mass index, calculated as weight (kg)/height (m)^2^; ID, internal diameter; ETT, endotracheal tube.

### 3.2 Correlation between documented intubation depth and predictive variables

**Figure 1:**
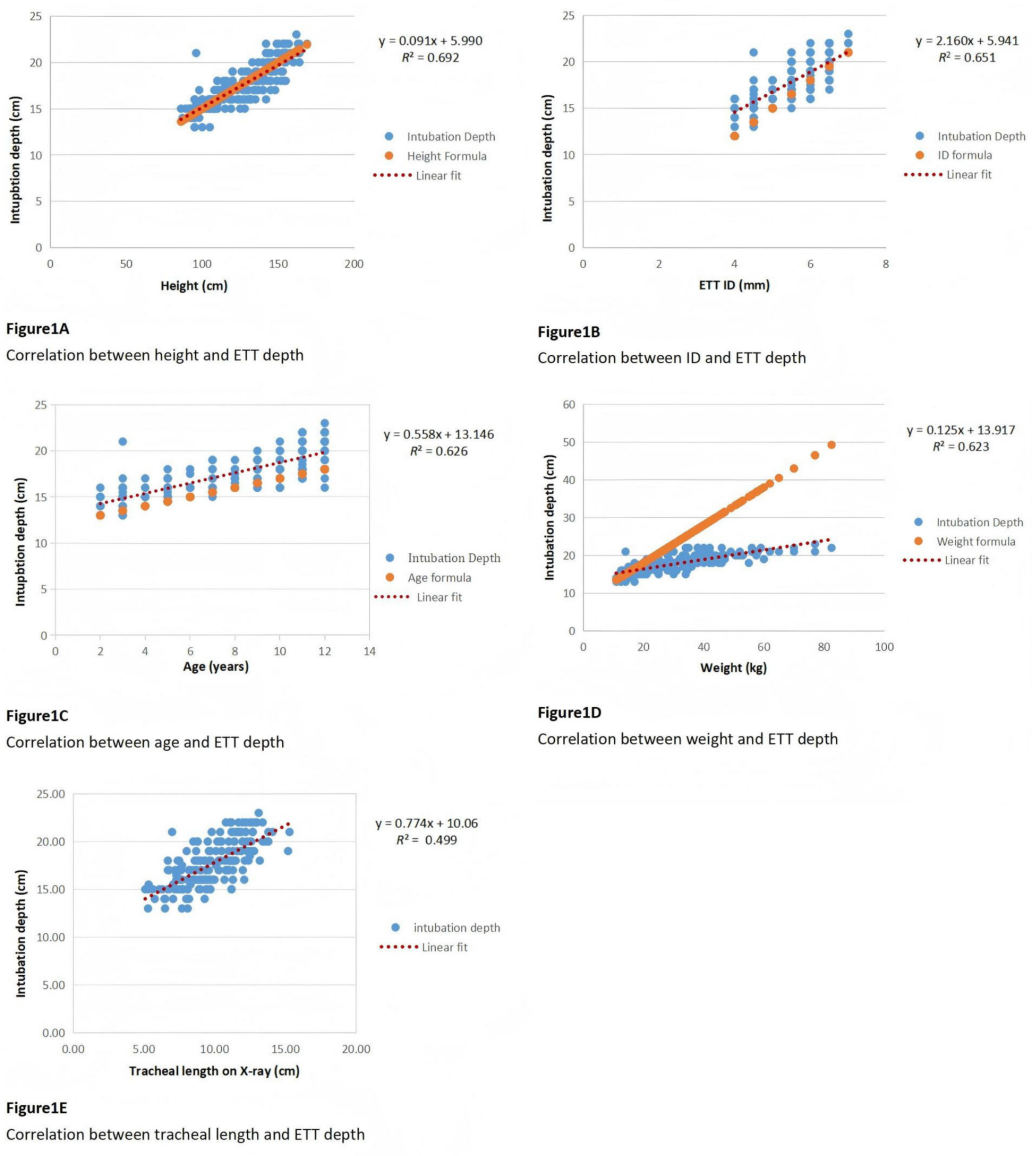
Regression curves and corresponding linear equations for each predictive variable.

All predictive factors showed a significant positive correlation with the documented ETT depth (Table 2). Height exhibited the strongest correlation (*r* = 0.832, *P* < 0.001) and explained the largest proportion of variance in documented ETT depth (*R*^2^ = 0.692). ID, age, and weight also demonstrated strong correlations with documented ETT depth (*r* = 0.807, 0.791, and 0.790, respectively; all *P* < 0.001). Although x-ray-measured tracheal length correlated relatively strongly with documented ETT depth (*r* = 0.706, *P* < 0.001), it yielded the lowest coefficient of determination (*R*^2^ = 0.499). Figure 1(A–E) Regression curves and corresponding linear equations for each predictive variable (Height, ETT ID, Age, Weight, and Tracheal length on X–ray, respectively), which were derived using the least squares method to model the linear relationship between each variable and documented ETT depth.

**Table 2:** Correlation between predictive variables and documented ETT depth (*N* = 197).

Predictive variable	R2	r	p
Height (cm)	0.692	0.832	< 0.001
ID (mm)	0.651	0.807	< 0.001
Age (years)	0.626	0.791	< 0.001
Weight (kg)	0.623	0.790	< 0.001
Tracheal length (cm)	0.499	0.706	< 0.001

ID, internal diameter.

### 3.3 Multivariable analysis of independent predictors

A stepwise multivariable linear regression analysis was performed to identify independent predictors of ETT depth. As shown in Table 3, height was the first variable to enter the model, accounting for 69.2% of the variance (*R*^2^ = 0.692). Weight and ID were then entered, resulting in a final model that is Height×0.047 + Weight  ×  0.037 + ID × 0.585 + 5.990. The final model achieved an adjusted *R*^2^ of 0.731 (*P* < 0.001), indicating that these three variables together explained 73.1% of the variance in documented ETT depth.

**Table 3:** Multivariable linear regression analysis of independent predictors of documented ETT depth (*N* = 197).

Variable	Unstandardized B	Standardized *β*	t	p	95% CI
Height (cm)	0.047	0.429	3.938	<0.001	0.024–0.071
Weight (kg)	0.037	0.233	2.934	0.004	0.012–0.062
ID (mm)	0.585	0.219	2.230	0.027	0.068–1.102

Stepwise selection method. Variables not retained: age, tracheal length. Adjusted *R*^2^ = 0.731, *p* < 0.001. Dependent variable: documented ETT depth. ID, internal diameter; ETT: endotracheal tube.

Height remained the strongest independent predictor In the final model (*β* = 0.429, *P* < 0.001), followed by weight (*β* = 0.233, *P* = 0.004) and ETT ID (*β* = 0.219, *P* = 0.027). Age and x-ray-measured tracheal length were excluded from the final model (*P* > 0.05).

### 3.4 Predictive performance of conventional formulas

Among the four conventional prediction formulas, the height-based formula (Height/10 + 5) achieved the lowest mean absolute error (MAE = 0.996 cm) and the highest safe proportion (72.08%), followed by the ID-based formula (MAE = 1.713 cm, safe proportion = 45.67%) and the age-based formula (MAE = 1.731 cm, safe proportion 44.67%). In contrast, the weight-based formula (Weight/2 + 8) exhibited a notably higher MAE of 5.418 cm and the lowest safe proportion (23.86%). Detailed error metrics and safety proportions are presented in Table 4.

**Table 4:** Error analysis and safe proportion of four predictive formulas compared to documented ETT depth.

Original formula	MAE (cm)	RMSE (cm)	Safe proportion (%)
Height-based (Height/10 + 5)	0.996	1.291	72.08
ID-based (ID × 3)	1.713	2.073	45.67
Age-based (Age/2 + 12)	1.731	2.143	44.67
Weight-based (Weight/2 + 8)	5.418	7.640	23.86

MAE, mean absolute error; RMSE, root mean square error; Safe proportion: percentage of patients for which |predicted depth-documented ETT depth| ≤ 0.15 × tracheal length. ID: internal diameter.

**Figure 2:**
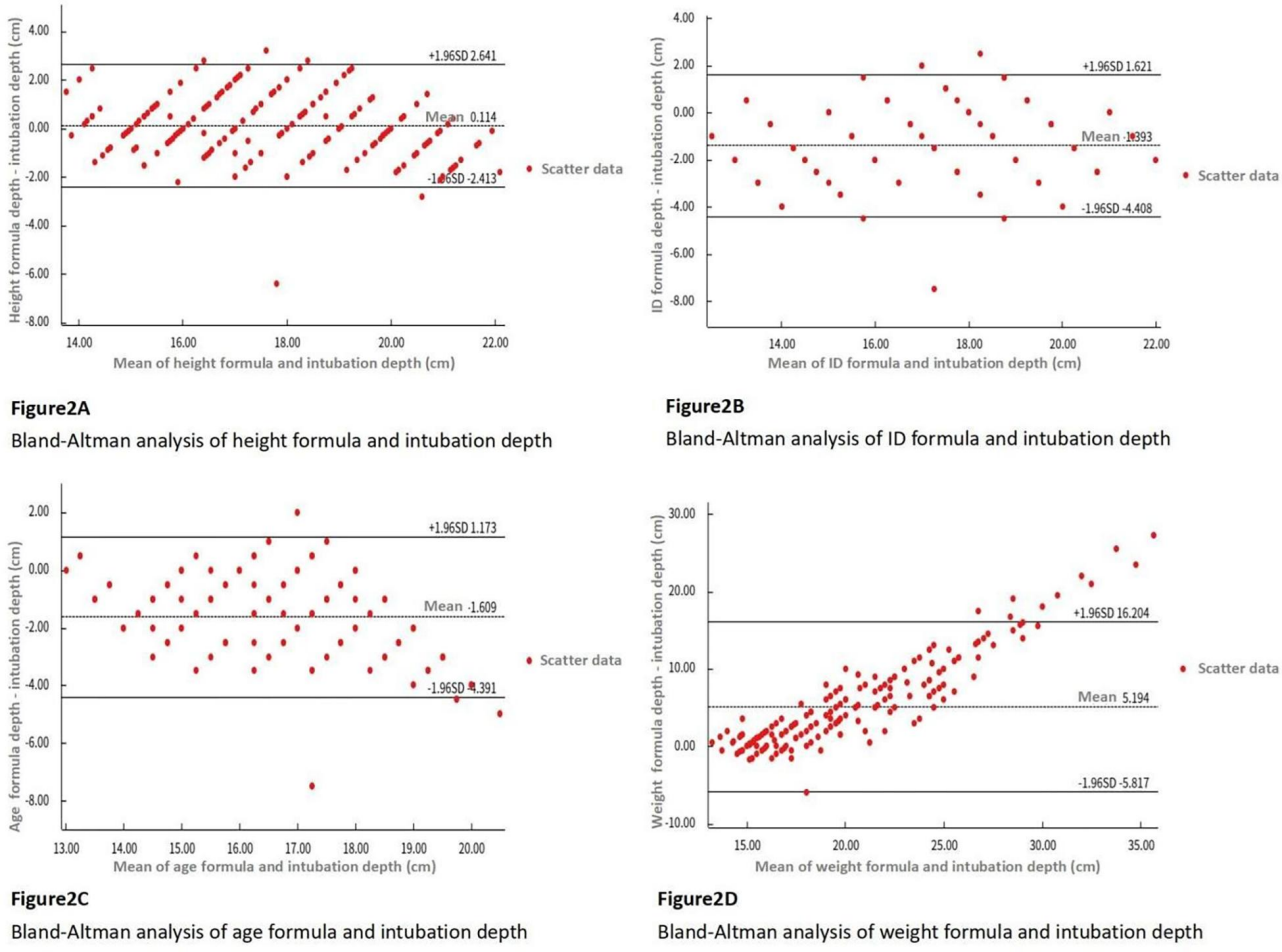
Bland-Altman analysis of four conventional predictive formulas.

Bland–Altman analysis was performed to assess the concordance between predicted and documented ETT depths (Figure 2). The height-based formula showed the smallest bias (0.114 cm) and the narrowest 95% limits of agreement (95% LoA: −2.413 to 2.641 cm), with data points evenly distributed around the zero line, indicating the best agreement (Figure 2A). The ID-based and age-based formulas exhibited biases of −1.393 cm and −1.609 cm, respectively, reflecting a systematic underestimation of approximately 1–2 cm (Figures 2B,C); both showed statistically significant bias and wide limits of agreement (*P* < 0.05). The weight-based formula presented the largest bias (5.194 cm) and the broadest 95% LoA (−5.817 to 16.204 cm), indicating poor predictive concordance (Figure 2D).

### 3.5 Optimization and evaluation of prediction formulas

Through linear regression and multivariable analysis, optimized prediction formulas were derived as follows:

Height-based regression: Height  ×  0.091 + 5.990 cm
ID-based regression: ID × 2.160 + 5.941 cm
Age-based regression: Age  ×  0.558 + 13.146 cm
Weight-based regression: Weight × 0.125 + 13.917 cm
Tracheal length-based regression: Length  ×  0.774 + 10.060 cm
Multivariable regression: Height  ×  0.047 + Weight × 0.037 + ID × 0.585 + 5.990 cm

**Figure 3:**
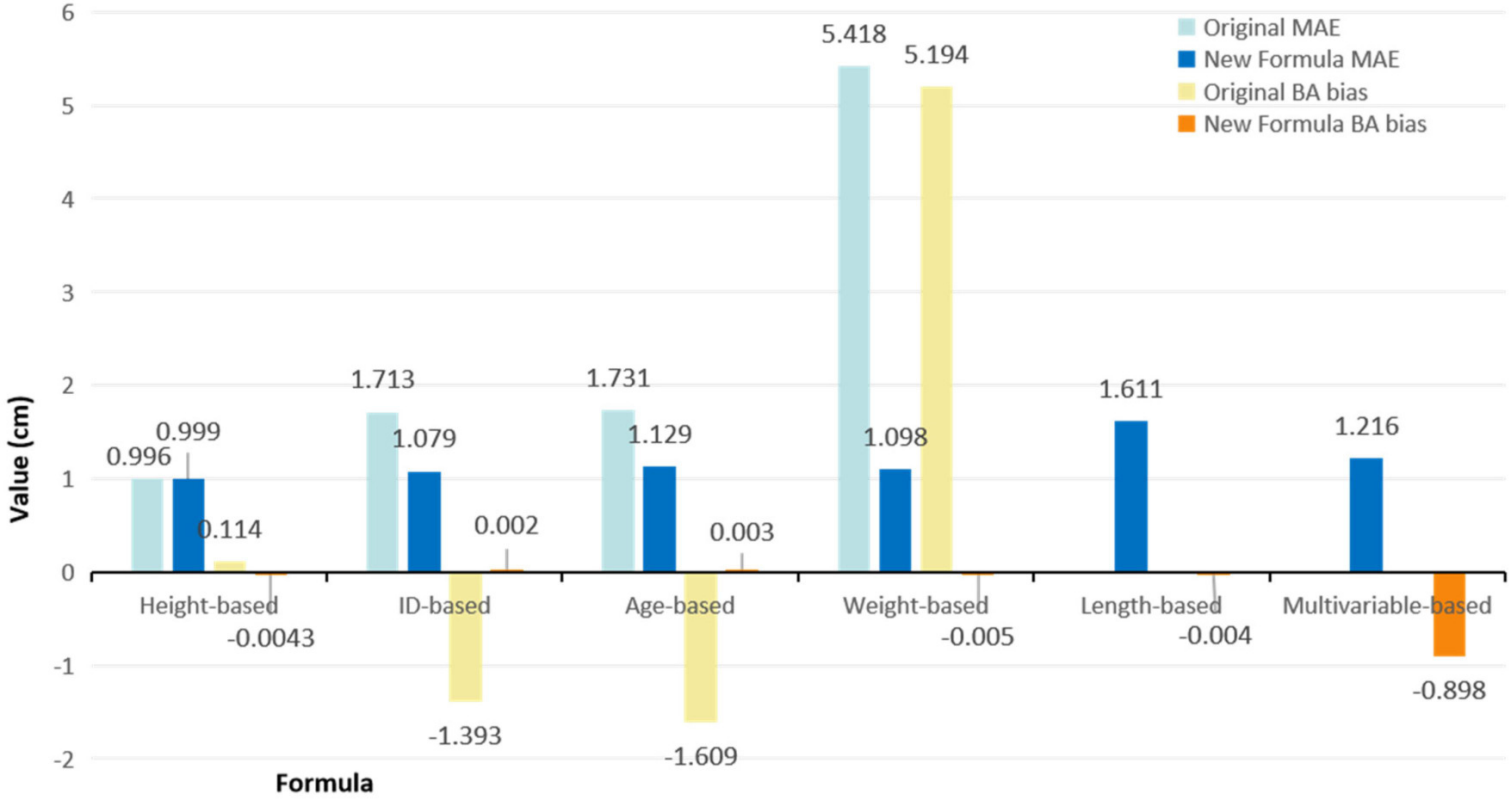
Bland-Altman analysis of optimized predictive formulas.

Five-fold cross-validation results (MAE, RMSE, safe proportion) and Bland-Altman analysis for these formulas are shown in Table 5. Compared with the original conventional formula, the optimized height-based regression showed a similar MAE (0.999 cm vs. 0.996 cm), similar safe proportion (72.23% vs. 72.08%), and reduced bias (–0.043 cm vs. 0.114 cm). The optimized age-based and ID-based regressions had lower MAEs (1.129 cm and 1.079 cm, respectively) and much improved safe proportions (69.8% and 64.7%, respectively) compared with the original formulas (MAE=1.731, 1.73 cm, safe proportion = 44.76, 45.67%). The weight-based regression showed the greatest improvement: MAE dropped from 5.418 cm to 1.099 cm, safe proportion increased from 23.9% to 71.2%, and bias was reduced to −0.005 cm. The tracheal length-based regression gave an MAE of 1.611 cm and safe proportion of 62.7%. The multivariable regression yielded an MAE of 1.216 cm and safe proportion of 67.1%. Figure 3 compares the MAE and bias between the original and each optimized formula. However, none of the optimized formulas outperformed the original height-based formula (Height/10 + 5).

**Table 5:** Five-fold cross-validation results for regression-derived formulas.

Optimized formula	MAE (cm)	RMSE (cm)	BA bias (cm)	Safe proportion (%)
Height-based regression	0.999	1.269	−0.043	72.23
ID-based regression	1.079	1.349	0.002	64.66
Weight-based regression	1.098	1.404	−0.005	71.20
Age-based regression	1.129	1.396	0.003	69.78
Tracheal length-based regression	1.611	1.626	−0.004	62.67
Multivariable regression	1.216	1.260	−0.898	67.13

BA bias: mean difference (predicted-documented ETT depth) from Bland-Altman analysi*s*. Safe proportion: percentage of patients for which |predicted depth- documented ETT depth | ≤ 0.15 × tracheal length.

Results of proportional bias analysis are summarized in Table 6. Significant proportional bias was present in most formulas, with the original weight-based and ID-based formulas showing positive bias and all regression-derived formulas showing negative bias.

**Table 6:** Proportional bias analysis for conventional and regression-derived formulas.

Formula	Slope (B)	95% CI	*p*
Height-based (Height/10 + 5)	−0.102	−0.187 to −0.017	0.019
Age-based (Age/2 + 12)	−0.379	−0.472 to −0.285	<0.001
ID-based (ID × 3)	0.126	0.034 to 0.218	0.007
Weight-based (Weight/2 + 8)	1.124	1.055 to 1.192	<0.001
Height-based regression (Height×0.091 + 5.990)	−0.204	−0.289 to −0.120	<0.001
ID-based regression (ID × 2.160 + 5.941)	−0.236	−0.327 to −0.145	<0.001
Weight-based regression (Weight×0.125 + 13.917)	−0.264	−0.359 to −0.168	<0.001
Age-based regression (Age × 0.558 + 13.146)	−0.260	−0.355 to −0.165	<0.001
Tracheal length-based regression (Length × 0.774 + 10.060)	−0.402	−0.515 to −0.289	<0.001
Multivariable regression (Height × 0.047 + Weight × 0.037 + ID × 0.585 + 5.990)	−0.123	−0.204 to −0.042	0.003

Slope (B) from linear regression of difference (predicted-documented ETT depth) on the mean of the two depths. Negative slope indicates overestimation in smaller children and underestimation in larger children; positive slope indicates the opposite.

## 4 Discussion

This study represents the first systematic evaluation of four commonly used pediatric ETT depth prediction formulas (Age/2 + 12, ID × 3, Height/10 + 5, Weight/2 + 8) and x-ray-measured tracheal length in children with scoliosis. Although height-based formulas are well-validated in children without spinal deformity, their applicability in this anatomically variant population remains unclear, and no prior study has comprehensively compared all four formulas or explored modifications for this cohort. Our findings address this gap by evaluating these formulas in a scoliosis population and, where applicable, providing optimized alternatives.

The findings indicate that within this anatomically variant population, the height-based formula “Height/10 + 5” showed the closest agreement with the clinically documented ETT depth, with a safe proportion of 72.08% (10). This observation is consistent with the strong correlation between height and documented depth (*r* = 0.832, *R*^2^ = 0.692), meaning that height alone explained nearly 70% of the variance in the recorded intubation depth. Notably, despite the presence of three-dimensional spinal deformity and thoracic structural alterations in this cohort, height—as a linear measure—maintained a proportional relationship with the total airway distance from the lips to the tracheal carina. This preserved relationship may be partly explained by findings from a CT-based study of tracheal anatomy, which suggested that height correlates primarily with cervical tracheal length rather than thoracic tracheal length (11). It is therefore possible that in children with scoliosis, the cervical trachea remains a midline structure whose longitudinal growth is relatively well reflected by standing height, whereas thoracic deformities do not substantially alter the vertical airwa*y* axis. Accordingly, height may serve as a useful reference for airway length in this patient population. Notably, the original height-based formula exhibited the smallest proportional bias (slope = −0.102) among all conventional formulas, suggesting relatively stable performance across different body sizes.

Consistent with the findings of Chinedu (12), the stability of the association between height and ETT depth may be attributed to the fact that height integrates the developmental status of multiple anatomical structures involved in airway length, making it less susceptible to distortion from localized spinal curvature compared to other anthropometric parameters (13). Another practical advantage of the height-based formula lies in the ready availability of height data (routinely collected in preoperative evaluations) and the simplicity of its calculation, which enhances its clinical applicability. Notably, a previous comparison of four predictive formulas in young children undergoing cardiac surgery also reported relatively high accuracy for the height-based method (14), further supporting its generalizability across different pediatric surgical populations. In our study, the height-based regression formula (Height × 0.091 + 5.990) showed little difference in MAE and safe proportion compared with the original height-based formula (Height/10 + 5), but its calculation is more complex and it is less easy to memorize. Moreover, simplifying it to a more memorable form (e.g., Height/10 + 6) would reduce predictive accuracy and introduce additional errors. Therefore, the regression-derived height formula offers no clear advantage over the original simpler formula.

In contrast to the height-based formula, the other three conventional predictive methods demonstrated notable limitations in this cohort. The widely cited age-based formula from the Advanced Pediatric Life Support (APLS) guidelines—Age/2 + 12—systematically underestimated the documented ETT depth, with an MAE of 1.731 cm, a bias of −1.609 cm, and a safe proportion of 44.67%. The linear regression-derived age formula (Age  ×  0.558 + 13.146) substantially improved performance, reducing the MAE to 1.129 cm, bias to 0.003 cm, and increasing the safe proportion to 69.78%, which is consistent with the findings of Lau's study (8). Although its safe proportion remained slightly lower than that of the height-based formula (72.08%), the optimized age formula achieved a clinically acceptable safety rate. Therefore, when height measurement is not available, this modified age formula may serve as a practical alternative, given its moderate correlation with documented depth (*r* = 0.791, *R*^2^ = 0.626).

The formula based on ETT ID (ID × 3) exhibited slope bias in our cohort, consistent with the Bland-Altman analysis results, which indicated that each 5 mm increase in tube diameter corresponded to an average increase in required depth of less than 3 cm. This is reflected in the original formula's bias of −1.393 cm, MAE of 1.713 cm, and safe proportion of 45.67%, with poor agreement with documented ETT depth (*P* < 0.05). Although the regression-optimized version (ID × 2.160 + 5.941) partially mitigated this bias (reduced to 0.002 cm) and improved accuracy (MAE = 1.079 cm), and increased the safe proportion to 64.66%, its MAE remained higher than that of the original height-based formula. This may be explained by the discrete and multifactorial nature of tube size selection, which is influenced not only by patient anatomy but also by clinician preference—a factor that introduces variability not accounted for in the formula. A previous prospective assessment of guidelines for determining appropriate ETT depth found that the correct placement rate was 75% when depth was calculated based on the actual ID of the catheter used, compared to 85% when calculated based on height (6), supporting the notion that ETT depth is more strongly influenced by height than by tube ID. Therefore, consideration should be given to the clinical criteria used for tube size selection when applying the ID-based formula in this patient population.

Weight exhibited considerable variability as a predictive parameter in this cohort. The original weight-based formula (Weight/2 + 8) showed a large bias (5.194 cm), wide 95% limits of agreement (−5.817 to 16.204 cm), a low safe proportion (23.86%), and a positive proportional bias (slope = 1.124, 95% CI: 1.055 to 1.192, *P* < 0.001), indicating a tendency to overestimate depth in larger children and underestimate it in smaller children. In contrast, the weight-based regression formula (Weight  ×  0.125 + 13.917) showed a bias of −0.005 cm, a safe proportion of 71.20%, and markedly improved agreement. These differences may be explained by the fact that weight distribution in children with scoliosis often differs from that of typically developing children (15), as scoliosis and associated thoracic deformities may alter body composition and reduce the correlation between weight and airway length. Thus, when height measurement is not available, the weight-based regression formula may serve as a secondary reference for this population.

The predictive value of directly measuring tracheal length using x-rays, which was hypothesized to be a strong predictor of intubation depth prior to this study, is worth discussing. Previous studies have used x-rays as a reference for verifying ETT placement and predicting ETT depth (16). In our cohort, x-ray-measured tracheal length showed a moderate correlation with documented ETT depth (*r* = 0.706, *P* < 0.001) and explained approximately 50% of the variance (*R*^2^ = 0.499). The tracheal length-based regression formula (Length  ×  0.774 + 10.060) demonstrated acceptable agreement, with a low systematic bias and a safe proportion of 62.67%, although its performance did not exceed that of the height-based formula. Several factors may contribute to this finding: radiographic measurement variability due to spinal rotation and thoracic deformity (common in scoliosis patients), the absence of clear subglottic anatomical landmarks on plain radiographs, and the discrepancy between the glottis-to-carina distance (measured on x-rays) and the clinically relevant incisor-to-carina length (used for intubation depth measurement) (17). These results suggest that tracheal length measured on plain radiographs may not be a strong independent predictor of ETT depth in children with scoliosis, but it could serve as a supplementary reference when clinically indicated.

In addition to the parameters mentioned above, depth markings on the tracheal tube are commonly used in clinical practice. In our institution, the initial ETT depth was routinely set at the second black line from the tube tip at the glottal level. This technique is convenient and rapid. However, the position of depth markings may vary among different manufacturers, requiring familiarity with the specific product's marking characteristics. After tube placement, accurately documenting the corresponding incisor marking is recommended to allow re-verification in case of tube displacement (18). Due to the retrospective design of this study, the relationship between the depth marking position and the final documented ETT depth was not systematically recorded; therefore, this method could not be quantitatively evaluated in our analysis, which is a limitation of the present study's scope.

This study provides preliminary, hypothesis-generating evidence for ETT depth prediction in children with scoliosis, a population with unique anatomical challenges. In this single-center cohort, the original formula “Height/10 + 5” showed the closest agreement with the clinically documented ETT depth. Because height is routinely measured in preoperative evaluations, this formula may serve as a reasonable initial guide. Furthermore, it exhibited the smallest proportional bias (slope = −0.102) among all formulas, indicating that its performance may be less influenced by body size. In cases where accurate height cannot be obtained, the regression-derived formulas from our study—weight-based regression (Weight  ×  0.125 + 13.917), age-based regression (Age  ×  0.558 + 13.146), and ID-based regression (ID × 2.160 + 5.941)—could be considered as secondary references. However, these optimized formulas lack external validation and were derived from the same dataset; therefore, they should be used with caution and only as exploratory estimates until prospectively validated in independent cohorts. Regarding the limits of agreement, even the best-performing formula (height-based) had a 95% LoA of approximately −2.4 to 2.6 cm. Given the mean tracheal length of 9.8 cm in our cohort, this degree of variation is clinically important and reinforces the need for post-intubation confirmation rather than reliance on any single formula.

All predicted depths should be regarded as initial estimates and must be confirmed at the bedside by auscultation of symmetrical breath sounds and capnography, consistent with the standard of care in our study. When feasible, fiberoptic bronchoscopy may be considered for direct visualization of tube position, particularly in children with severe deformities (such as a large Cobb angle or severe thoracic distortion) or complex syndromes associated with these deformities (such as Marfan syndrome), where individualized airway assessment is especially important (19).

Several limitations of this study should be acknowledged. First, the single-center, retrospective design introduces selection bias, and the findings may not be directly generalizable to other populations or settings. Second, the reference standard for documented ETT depth was determined by the attending anesthesiologist based on auscultation and capnography, rather than direct visualization via fiberoptic bronchoscopy, the current gold standard. Although this approach reflects routine clinical practice and no intraoperative tube malposition was recorded, some variability in depth measurement cannot be excluded, and potential incorporation bias cannot be completely ruled out despite the use of a standardized depth marking technique. Third, scoliosis-specific anatomical parameters (e.g., Cobb angle, apex vertebral position, deformity severity) were not systematically available in the medical records; therefore, subgroup analyses based on these factors could not be performed, limiting our ability to assess whether the findings apply uniformly across different types or severities of scoliosis. Nevertheless, all included children met surgical criteria for scoliosis correction (i.e., conservative treatment had failed and the deformity was clinically significant). Thus, our findings are likely applicable to this specific surgical population, although generalization to mild scoliosis or non-thoracic curves requires caution. Fourth, the optimized formulas (height-based regression, age-based regression, weight-based regression, ID-based regression, tracheal length-based regression, and multivariable regression) were derived and internally evaluated on the same dataset. Although five-fold cross-validation suggested minimal overfitting (the cross-validated MAEs were very close to the whole-sample MAEs), these formulas still lack external validation. Consequently, they should be considered exploratory and require prospective validation in independent cohorts. Collectively, these limitations suggest that our findings are most applicable to a relatively homogeneous population of children with scoliosis who are candidates for posterior thoracolumbar corrective surgery. Future multicenter prospective studies incorporating comprehensive deformity parameters and advanced imaging technologies——such as low-dose computed tomography (CT)——would help develop more accurate and personalized predictive models.

## 5 Conclusion

In this single-center retrospective study of 197 children aged 2–12 years with scoliosis, the classic height-based formula “height/10 + 5” showed the closest agreement with the clinically documented ETT depth, with a mean absolute error of 0.996 cm and a safe proportion of 72.08%. The commonly used age formula (age/2 + 12) exhibits systematic underestimation, whereas the internal diameter formula (ID × 3) shows slope bias. The regression-derived formulas (age-based regression: Age  ×  0.558 + 13.146; ID-based regression: ID × 2.160 + 5.941; weight-based regression: Weight×0.125 + 13.917) demonstrated improved agreement compared with their original versions, but none surpassed the original height-based formula in this cohort. The original weight-based formula (weight/2 + 8) performed poorly and is not recommended. Tracheal length measured on plain radiographs explained only about 50% of the variance in documented ETT depth and should not be used as a sole predictor; it may serve as a supplementary reference in selected circumstances. All findings are hypothesis-generating, and the regression-derived formulas lack external validation. Therefore, any predicted depth should be confirmed at the bedside, and prospective studies are needed to validate these results before clinical adoption.

## Data Availability

The data analyzed in this study is subject to the following licenses/restrictions: Reasonable requests for access to the de-identified data may be considered by the corresponding author, subject to institutional approval and a formal data sharing agreement. Requests to access these datasets should be directed to Name: Bo Zhu, zhubo@pumch.cn
